# Single nucleotide variants in immune-response genes and the tumor microenvironment composition predict progression of mantle cell lymphoma

**DOI:** 10.1186/s12885-021-07891-9

**Published:** 2021-03-01

**Authors:** Guilherme Rossi Assis-Mendonça, André Fattori, Rafael Malagoli Rocha, Gustavo Jacob Lourenço, Márcia Torresan Delamain, Suely Nonogaki, Vladmir Cláudio Cordeiro de Lima, Gisele Wally Braga Colleoni, Cármino Antonio de Souza, Fernando Augusto Soares, Carmen Silvia Passos Lima, José Vassallo

**Affiliations:** 1 Department of Pathology, Faculty of Medical Sciences, University of Campinas, Distrito de Barão Geraldo, Campinas, SP Brazil; 2 Department of Internal Medicine, Faculty of Medical Sciences, University of Campinas, Campinas, SP Brazil; 3 Molecular Gynecology Laboratory, Department of Gynecology, Federal University of São Paulo, São Paulo, Brazil; 4 Laboratory of Cancer Genetics, Faculty of Medical Sciences, University of Campinas, Campinas, SP Brazil; 5 Hematology and Hemotherapy Center, University of Campinas, Campinas, Brazil; 6 Instituto Adolfo Lutz, Secretaria de Estado da Saúde, São Paulo, SP Brazil; 7 Department of Medical Oncology, A C Camargo Cancer Center, São Paulo, Brazil; 8 Department of Clinical and Experimental Oncology, Federal University of São Paulo, São Paulo, SP Brazil; 9 Rede D’Or Hospitals Network - Pathology Division, São Paulo, SP Brazil; 10 Laboratory of Investigative and Molecular Pathology (LIP), CIPED, Faculty of Medical Sciences, University of Campinas, Campinas, SP Brazil

**Keywords:** Mantle cell lymphoma, Tumor microenvironment, SNVs, Immunohistochemistry, Prognostic factors

## Abstract

**Background:**

There is evidence to consider that the tumor microenvironment (TME) composition associates with antitumor immune response, and may predict the outcome of various non-Hodgkin lymphoma subtypes. However, in the case of mantle cell lymphoma (MCL), a rare and aggressive disease, there is lacking a detailed study of the TME components, as well as an integrative approach among them in patients’ samples. Also, from the genetic point of view, it is known that single nucleotide variants (SNVs) in immune-response genes are among important regulators of immunity. At present, it is uncertain whether SNVs in candidate immune-response genes and the TME composition are able to alter the prognosis in MCL.

**Methods:**

We assessed a detailed TME composition in 88 MCL biopsies using immunohistochemistry, which was automatically analyzed by pixel counting (Aperio system). We also genotyped SNVs located in candidate immune-response genes (*IL12A*, *IL2*, *IL10*, *TGFB1*, *TGFBR1*, *TGFBR2*, *IL17A*, *IL17F*) in 95 MCL patients. We tested whether the SNVs could modulate the respective protein expression and TME composition in the tumor compartment. Finally, we proposed survival models in rituximab-treated patients, considering immunohistochemical and SNV models.

**Results:**

High FOXP3/CD3 ratios (*p* = 0.001), high IL17A levels (*p* = 0.003) and low IL2 levels (*p* = 0.03) were individual immunohistochemical predictors of poorer survival. A principal component, comprising high quantities of macrophages and high Ki-67 index, also worsened outcome (*p* = 0.02). In the SNV model, the CC haplotype of *IL10* (*p* < 0.01), the GG genotype of *IL2* rs2069762 (p = 0.02) and the AA+AG genotypes of *TGFBR2* rs3087465 (*p* < 0.01) were independent predictors of outcome. Finally, the GG genotype of *TGFB1* rs6957 associated with lower tumor TGFβ levels (*p* = 0.03) and less CD163+ macrophages (*p* = 0.01), but did not modulate patients’ survival.

**Conclusions:**

Our results indicate that the TME composition has relevant biological roles in MCL. In this setting, immunohistochemical detection of T-reg cells, IL17A and IL2, coupled with SNV genotyping in *IL10*, *TGFBR2* and *IL2,* may represent novel prognostic factors in this disease, following future validations.

**Supplementary Information:**

The online version contains supplementary material available at 10.1186/s12885-021-07891-9.

## Background

Mantle cell lymphoma (MCL) is an uncommon non-Hodgkin lymphoma (NHL) subtype, marked by presence of the t (11:14) translocation in more than 90% of the cases, which leads to overexpression of cyclin D1 [1]. MCL has an aggressive clinical course, debilitating potential and yet limited prognostic stratification. Some features are already known to impact MCL survival, such as the use of rituximab (anti-CD20 antibody) and the Mantle Cell Lymphoma International Prognostic Index (MIPI). The Ki-67 cell proliferation index, assessable in patients’ biopsies, is also proposed as directly related to tumor aggressiveness [2]. More recently, molecular events, such as B-cell receptor activation and *TP53* / *CDKN2A* mutations, were associated with treatment resistance [3, 4]. These results brought new insights in the pathophysiology of MCL. However, factors such as the interactions between tumor cells and surrounding inflammatory populations need further exploration.

In this setting, the composition of tumor microenvironment (TME) has emerged as a promising prognostic marker in patients with a variety of tumors, including NHLs [5]. The TME encompasses extracellular matrix, inflammatory cells, fibroblasts, the vascular bed and soluble signaling molecules. Interactions among these components control oxygen and nutrient supplies for tumor cells, and also regulate the antineoplastic immune response [5]. Depending on the balance among TME components, disease course may be defined either as immune evasion and tumor progression, or as an efficient immune response and disease clearance [6]. Assessment of the TME cellular composition has helped to better stratify prognosis in various types of NHLs [5]. For MCL, the roles of circulating monocytes [7], T-cells [8] and follicular dendritic cells [9] on patients´ outcome have been demonstrated in few studies, but the remaining inflammatory cells, as well as an integrative approach among them, remain largely unexplored in this disease. Moreover, inflammatory cytokines, which compose the molecular counterpart of the TME, are also increasing subjects of interest in lymphoma, due to their capacity of modulating immune responses and lymphoma cells’ growth [10–13]. For instance, one recent study characterized, in vitro, the role of IL10 in maintaining survival of MCL cells via M2-macrophages [13]. These findings not only highlighted important interplays among MCL cells, TME cells and cytokines, but also stressed the need of exploring cellular and molecular parameters of the TME in patient-derived samples.

However, as the capacity of immune response is variable in humans [14], it may be relevant to assess not only the levels of cytokine profiles, but also the genetic determinants for their expression. Previous studies demonstrated that single nucleotide variants (SNVs) located in immune-response genes, including cytokines, may alter NHL onset and progression [14–17]. This may be especially valid in the case of functional variants, in which alteration of the transcript and/or protein may regulate the tumor microenvironment composition and ultimately modulate disease outcome [18]. However, this question was not yet properly addressed in MCL, despite the role of immune subsets in sustaining the survival of lymphoma cells [11, 13, 19].

Herein, we assessed the prognostic role of immune-response components of the TME in biopsies from a retrospective cohort of MCL. We also studied SNVs in immune-response related genes, attempting to elucidate whether they could alter the TME composition and the outcome of MCL patients.

## Methods

### Patient selection and clinical data

We analyzed all 122 MCL cases diagnosed between 1999 and 2016 at the Hematology and Hemotherapy Center of the University of Campinas (*n* = 74) and A. C. Camargo Cancer Center (*n* = 48).

The diagnosis of MCL was made according to the World Health Organization (WHO) Classification for Lymphoid Tumors [20]. Tumor cells were characterized by a CD20+/CD5+/cyclin D1+ phenotype. The Mantle Cell International Prognostic Index (MIPI) was calculated and used as the reference prognostic instrument [2].

### Single genetic variants choice and genotyping

The choice of SNVs was based on a candidate gene approach. We selected SNVs in genes related to immune-response with previous evidences of active roles in lymphoma, cancer or modulation of the immune response [13–16, 21–42]. A minor allele frequency (MAF) of 5% was preconized. Sixteen SNVs were finally selected in 8 candidate genes: *IL12A*, *IL2*, *IL10*, *TGFB1*, *TGFBR1*, *TGFBR2*, *IL17A* and *IL17F* (Table 1).

**Table 1:** Genes, single nucleotide variants and their biological rationale for inclusion in this study

Gene	SNVs	Biological rationale
***IL12A***	rs755004, rs485497, rs568408, rs583911	-Controversial molecule in B-cell lymphoma models (antitumoral effect / exhaustive effect in T-cells) [21, 22]; -SNVs previously associated with lymphoproliferative disorders [16, 23–25].
***IL2***	rs2069762, rs6822844	-Cytokine with potential cytotoxic effect in mantle cell lymphoma [26]; -SNVs previously associated with the regulation of IL2 levels and lymphoma prognosis [15, 27].
***IL10***	rs3024491, rs1800872, rs1800890	-Cytokine with effects on mantle cell lymphoma proliferation and survival [13, 28]; -SNVs were previously implicated on regulation of IL10 levels and lymphomagenesis [14, 29–31].
***TGFB1***	rs6957, rs1800471, rs1800469	-Pathway with a potential role in mantle cell lymphoma signaling [32]; -SNVs associated with functional changes in the TGFβ pathway [33–35].
***TGFBR1***	rs334348	-Pathway with a potential role in mantle cell lymphoma signaling [32]; -SNV with an effect on cancer risk and postulated functional change [36].
***TGFBR2***	rs3087465	-Pathway with a potential role in mantle cell lymphoma signaling [32]; -SNV associated with changes in promoter activity [37].
***IL17A***	rs3748067	-Molecule with potential but still uncertain role in B-cell lymphomas’ pathophysiology, including mantle cell lymphoma [38, 39]; -SNV previously associated with prognostic features in cancer, and with a putative functional role [40].
***IL17F***	rs763780	-Molecule with a potential role in B-cell lymphomas, and adverse prognostic role in T-cell lymphoma [38, 39, 41]; -SNV previously associated with alterations in protein function [42].

*SNVs* Single nucleotide variants

DNA samples were extracted from peripheral blood of patients, using precipitation with lithium chloride. Assessments of DNA yield (ng/μL) and purity (260/280 and 260/230 ratios) were performed using the NanoDrop spectrophotometer (ThermoFisher Scientific, Wilmington, DE, USA). Whenever necessary, sodium acetate (3 M) was added to the extracted DNAs, followed by new ethanol precipitations, to improve purity. The final concentration of all samples was set to 50 ng/μL.

Genotyping of SNVs was performed using the Taqman® OpenArray® QuantStudio™ Real-Time PCR System (Life Technologies Inc., Carlsbad, CA, USA). Briefly, DNA samples were pipetted together with Taqman® Openarray® Master Mix on 384-well plates. This mixture was then transferred to genotyping plates using the Openarray® Accufill™ system. Thermocycling was performed during 40 cycles, and visualization of polymorphic alleles was possible by using fluorophores (VIC™ and FAM™). A single reaction allowed the simultaneous detection of all 16 SNVs.

### Tissue samples and immunohistochemistry

Formalin-fixed, paraffin embedded (FFPE) diagnostic blocks from MCL cases were obtained from the participating hospitals. All slides were reviewed by an experienced hematopathologist (JV), and two representative areas from each source block were selected to construct a tissue microarray (TMA). Core fragments with diameter of 1 mm were taken using a Tissue Microarrayer (Beecher Instruments, Silver Springs, MD), and samples were put as duplicates in the recipient block. Immunohistochemistry was then performed, using a broad panel of antibodies to study key TME components, including tumor-infiltrating lymphocytes (CD3, CD4, PD1, FOXP3, CD8, granzyme B, perforin, CD57) and macrophages (CD68, CD163, iNOS). We also performed immunohistochemistry to detect the proteins encoded by the genes used in the SNV approach (IL12A, IL2, IL10, TGFβ, TGFBR1, TGFBR2, IL17A, IL17F). Reactions for Ki-67 and SOX11 were performed as well. A detailed list of antibodies, including suppliers and dilutions, is in supplementary Table 1.

Eighty-nine blocks were used for SOX11 evaluation, and 88 were considered suitable for TME assessment.

The immunohistochemical (IHC) reaction was performed using standard procedures. Briefly, unstained slides were submitted to antigen retrieval using citric acid solution/pH 6.0 or EDTA/pH 9.0 buffers. Endogenous peroxidase activity was blocked by hydrogen peroxide solution for 20 min. Exposure to the primary antibody was performed overnight. The signal was amplified by a third generation polymer tagged with anti-mouse/anti-rabbit immunoglobulins and horseradish peroxidase (Novolink Polymer Detection System, Leica Biosystems, Newcastle Upon Tyne, UK), and the color was developed with diaminobenzidine chromogenic substrate (Sigma, D5637, St. Louis, MO, USA). Positive cells were observed in golden brown color. The negative control was performed by omitting the primary antibody.

### Immunohistochemical analysis

**Fig. 1:**
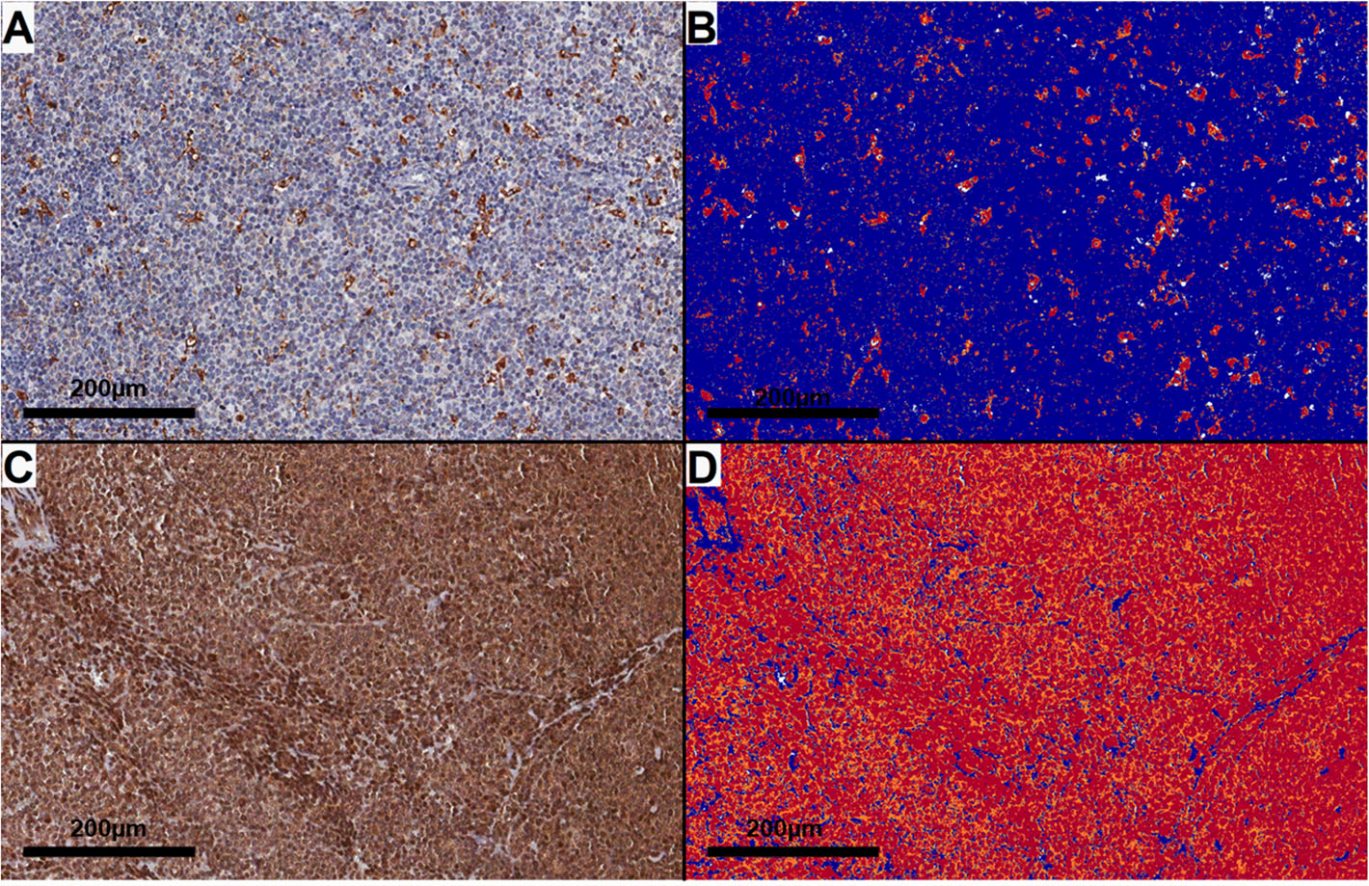
Representative examples of immunohistochemical analysis using the Aperio system in mantle cell lymphoma. Assessment of CD68 (**a**-**b**) and IL17A (**c**-**d**), in which “**a**” and “**c**” illustrate the original stainings, and “**b**” and “**d**”, the respective decodified images. In the latter ones, blue color identifies negative pixels, yellow color indicates weak positive staining, orange color highlights positive staining and red color denotes strong positive staining

Quantification of IHC markers was performed on the entire TMA cores. For the majority of the markers, this was done automatically using the Aperio ImageScope™ software (Leica Microsystems Inc., Buffalo Grove, IL, USA) (Fig. 1). The Positive Pixel Count algorithm was used to grade pixels as negative, low positive, positive and high positive. Inputs for the algorithm were a hue value of 0.1, hue width of 0.5 and color saturation threshold of 0.1 (for most cores). In some rare cases presenting with nonspecific background, the color saturation threshold was increased to 0.15 to minimize noise capture. For antibodies staining specific TME populations (e.g. CD68, FOXP3, CD3), the fraction of all positive pixels was considered as the score. For antibodies that heterogeneously stained both tumor and microenvironment cells (e.g. cytokine antibodies), we calculated the H-score, which applies different multipliers for low positive, positive and high positive stainings (1, 2 and 3, respectively) [43].

For SOX11 assessment, we performed categorization using a visual approach, similarly to others [44, 45]. In this setting, cases were classified as SOX11^high^ (nuclear staining in more than 10% of cells) or SOX11^low^ (nuclear staining in less than 10% of cells or negative staining).

Immunohistochemical scores were analyzed individually and also as part of a dimension-reducing methodology (principal component analysis; please see “statistical analyses”).

### Statistical analyses

Associations between variables were assessed using χ2, Fisher’s exact test, Mann Whitney’s test and Spearman’s correlation index. When necessary, continuous variables were dichotomized as “high” and “low” based on the median values.

Regarding SNVs assessment, the Hardy-Weinberg equilibrium (HWE) was evaluated using the chi-square (χ2) goodness-of-fit test. Pairwise linkage disequilibrium (LD) analyses were performed using the Haploview 4.2 software (www.broad.mit.edu/mpg/ haploview) to ensure that the markers were appropriate for inclusion in the haplotype estimates. The LD was measured by the disequilibrium coefficient (D′), and LD significance was considered at a D′ ≥ 80%.

Exploratory principal component analysis (PCA) with Varimax rotation was used as a dimension-reducing method in IHC quantifications. Interactions between IHC variables were estimated and expressed as principal components. In this setting, stronger interactions had higher computed variance values [46]. The associations between principal components and clinicopathological features were estimated with linear regression analyses.

Survival analyses were also performed. However, as anti-CD20 therapy may be a potential confounder for the TME function, only patients who received rituximab in first-line regimens were included in this analysis [47]. In a similar way, only patients that did not undergo bone marrow transplantation were put in survival analysis, as transplantation modulates the proportion of immune cells [48, 49]. Overall survival (OS) was defined as the time from diagnosis until death by any cause or last follow-up. Event-free survival (EFS) was defined as the time from diagnosis until death, disease progression or last follow-up. Three survival models were tested: one model considering individual IHC assessments (IHC model), another one testing genotyping data (SNV model), and one last model using the data from PCA. Survival curves were plotted with the Kaplan-Meier method, and compared using the log-rank test. We further performed Cox univariate regressions for variables influencing survival in Kaplan-Meier curves. Finally, a Cox multivariate model was proposed, enclosing all variables with a *p* value of less than 0.10 in the univariate analysis. Simultaneous testing of redundant information (e.g. age and the MIPI) was not performed. Follow-up update was performed on January, 2019.

The significant results of multivariate Cox regressions were internally validated using the Bootstrap resampling method (1000 replications).

When necessary, the Benjamini-Hochberg method was performed for correction of multiple comparisons. All statistical tests were two-tailed, and a *p* value of less than 0.05 was considered statistically significant.

## Results

### Demographic and clinical characteristics of the population

Median age at diagnosis for all MCL patients was of 66 years old (range: 31–93), and there was a predominance of male sex (93/122 or 76.2%). The majority of patients (107/122 or 87.7%) were included in Ann Arbor stages III or IV. The MIPI allowed classification of available patients as high-risk disease (35.3%), followed by intermediate risk (27.0%) and, finally, low risk (24.6%). Seventy-eight patients (63.9%) had received rituximab as first-line therapy. Twenty-seven patients (22.1%) underwent hematopoietic stem-cell transplantation (autologous in 26 cases and allogeneic in 1). The main clinical variables are detailed in supplementary Table 2.

### Immunohistochemical quantifications

**Fig. 2:**
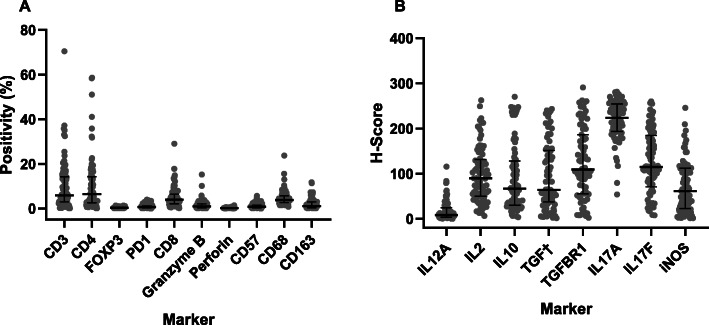
Quantifications of the tumor microenvironment markers in mantle cell lymphoma. **a** Markers quantified using positivity levels (0–100%) and (**b**) Markers quantified using the H-Score (0–300). Graphs show scatterplots with superimposed median levels and interquartile range

In 88/122 (72.1%) cases, diagnostic FFPE blocks were suitable for TME assessment. The most frequent immune cells found on the MCL biopsies were T lymphocytes, mainly CD4+ (median = 6.43% of pixels, range: 0.09–58.60). Macrophages (CD68+) were slightly less frequent (median = 3.82% of pixels, range: 0.79–23.69). Regarding inflammatory cytokines, IL17A had the highest expression (median HScore = 223.85, range: 54.18–281.40) (Fig. 2). The expression of TGFBR2 was visually negative in all cases, therefore, no quantification was performed. Representative stainings for each marker are available in supplementary Figs. 1, 2 and 3.

**Fig. 3:**
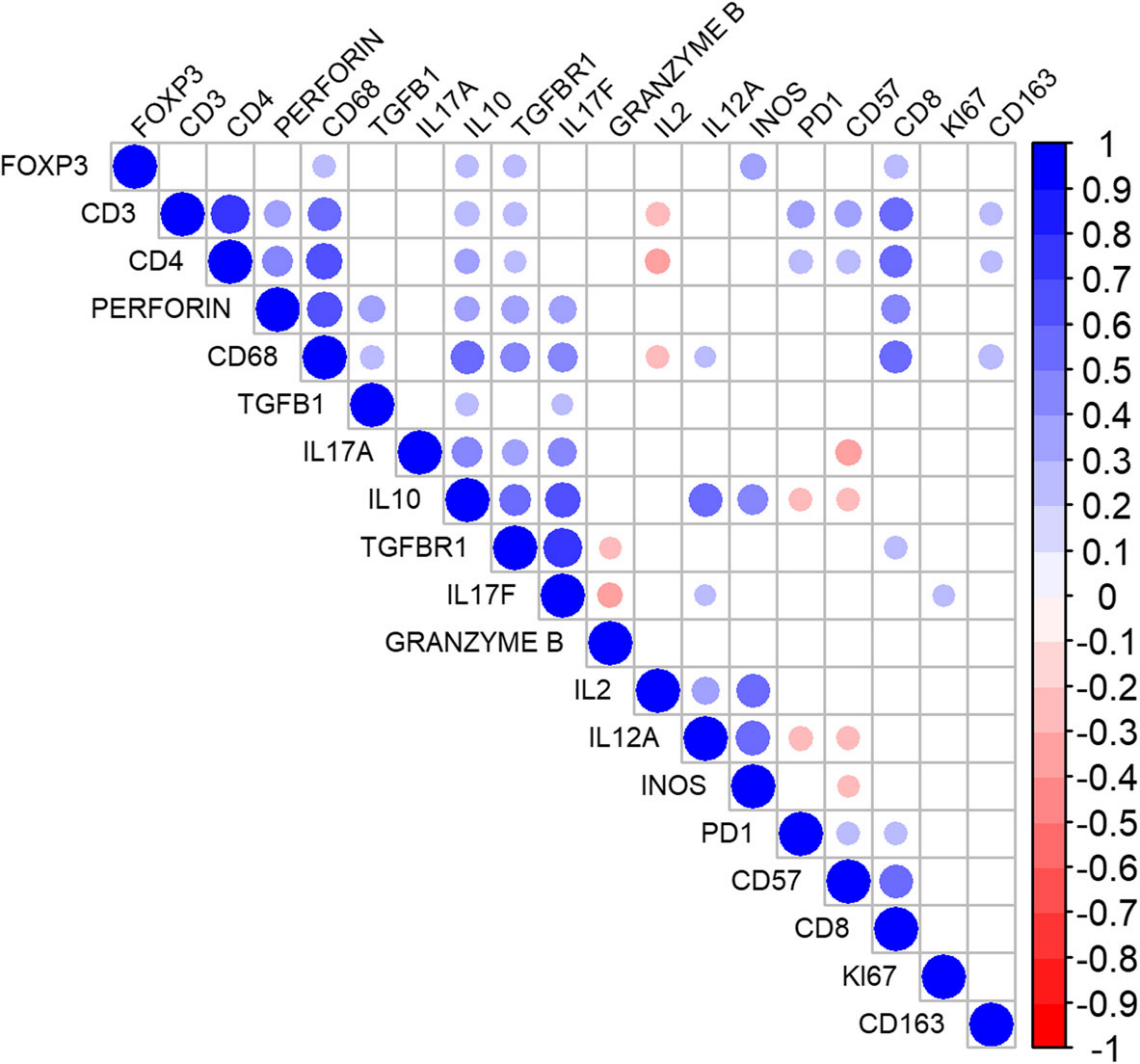
Correlation matrix for immunohistochemical markers in mantle cell lymphoma. Blueish tones indicate higher positive correlations, whereas reddish tones point toward negative correlations. Larger circle diameters denote higher modules of the correlation coefficient (r). Non-significant correlations are shown as white intersections

Positive correlations were found between some of the IHC markers: CD4 with CD8 (*p* < 0.001), perforin with CD8 (*p* = 0.02), and CD68 with CD4 (p < 0.001), CD8 (*p* = 0.001), and IL10 (p < 0.001). IL17A and CD57 were inversely correlated (*p* = 0.008) (Fig. 3).

Higher pixel counts of PD1+ cells were associated with less B-symptoms at diagnosis (*p* = 0.04). Also, higher levels of macrophages (CD68+) and higher CD4/CD8 ratios were found in patients with less aggressive MIPI categories (low or intermediate risks, *p* = 0.02 and 0.04, respectively). In contrast, higher CD8/CD3 ratios were more frequently found on high-risk MIPI patients (p = 0.02). Finally, high IL12A and IL17A levels associated, respectively, with bone marrow infiltration (*p* = 0.01) and blastoid cytology (*p* = 0.04) (supplementary Tables 3 and 4).

Regarding SOX11 evaluation, the majority of patients were categorized as SOX11^high^ (76 out of 89 evaluable cases, or 85.4%). The remaining cases (13/89 or 14.6%) were classified as SOX11^low^ (representative photomicrographs in supplementary Fig. 4). There was a higher percentage of female patients in the SOX11^low^ group (46.2 vs 19.7%; *p* = 0.03); no other clinicopathological differences were seen (supplementary Table 5). Composition of the TME was mostly similar between SOX11^high^ and SOX11^low^ groups, except for a trend towards higher IL2 and perforin levels in SOX11^low^ cases (supplementary Fig. 5).

### Single nucleotide variants genotyping and TME composition

Genotyping of SNVs was possible in 95 patients. In all but 1 SNV (*TGFBR1* rs334348) the HWE was observed (supplementary Table 6).

**Fig. 4:**
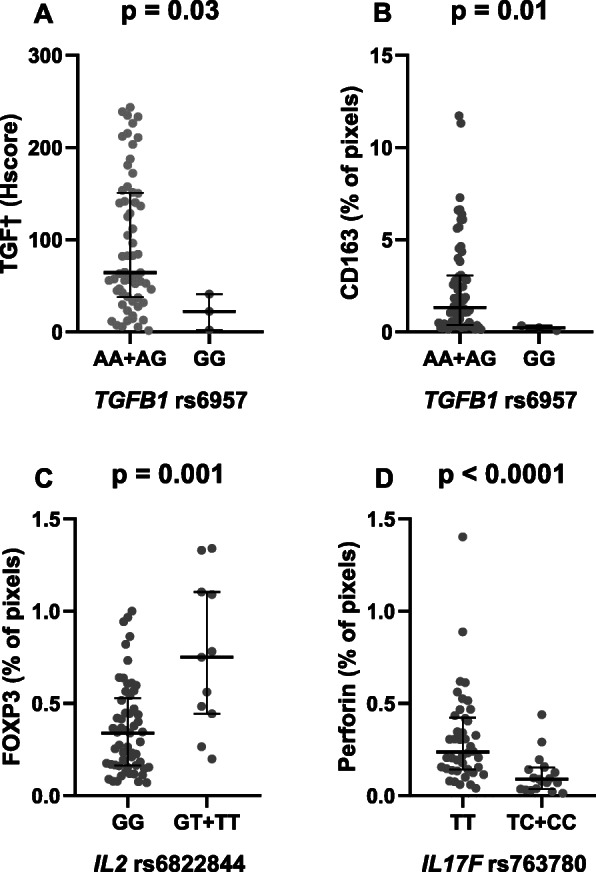
Modulation of the tumor microenvironment in mantle cell lymphoma by single nucleotide variants. Associations of *TGFB1* rs6957 with (**a**) TGFβ and (**b**) CD163 levels. **c** Association of *IL2* rs6822844 with FOXP3 levels; (**d**) Association of *IL17F* rs763780 with perforin levels. Graphs show scatterplots with superimposed median levels and interquartile range. All *p*-values were obtained with two-tailed Mann-Whitney tests

The only SNV associated with the respective protein levels was *TGFB1* rs6957. Significantly lower TGFβ levels were found in tumors from patients harboring the GG genotype (*p* = 0.03, Fig. 4a). The same genotype also associated with decreased CD163+ pixels (*p* = 0.01, Fig. 4b).

Besides, the recessive “T” allele of *IL2* rs6822844 was seen in patients with increased FOXP3 pixel counts (*p* = 0.001, Fig. 4c). Finally, considering *IL17F* rs763780, patients carrying the recessive “C” allele had tumors with lower quantities of perforin-expressing cells (*p* < 0.0001, Fig. 4d).

Based on the LD coefficients, we were able to estimate haplotypes in *IL12A* and *IL10* (supplementary Table 7, supplementary Fig. 6). The haplotypes presented no associations with features at diagnosis (data not shown).

### Survival models including individual immunohistochemical markers and SNVs

Survival analyses were performed in 55 patients that received rituximab and did not undergo stem cell transplantation. Univariate analyses considering clinical features evidenced significant adverse prognostic roles of B-symptoms (both in EFS and OS), bone marrow infiltration (OS only) and MIPI index (trend of significance in EFS only (supplementary Table 8). These variables were, therefore, used as covariates on multivariate Cox regressions.

**Fig. 5:**
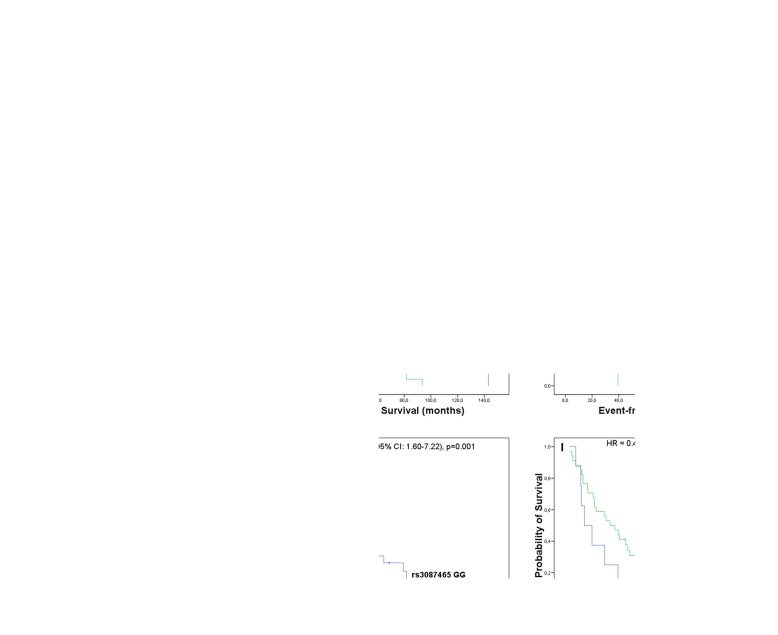
Kaplan-Meier curves (event-free survival) of mantle cell lymphoma patients. Categorizations by (**a**) IL2 levels, (**b**) FOXP3/CD3 ratios, (**c**) CD8/CD3 ratios, (**d**) Granzyme B levels, (**e**) SOX11 positivity, (**f**) Genotypes of *IL2* rs2069762, (**g**) Haplotypes in *IL10*, (**h**) Genotypes of *TGFBR2* rs3087465 and (**i**) Genotypes of *IL17F* rs763780. Each graphic contains the hazard ratios and *p*-values obtained in the respective univariate Cox regressions

**Fig. 6:**
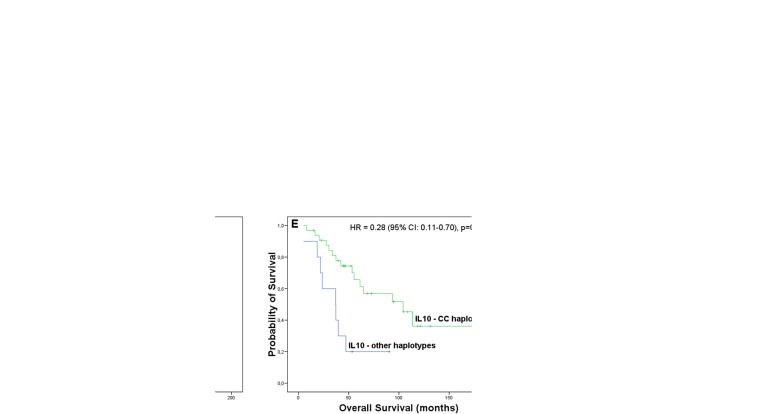
Kaplan-Meier curves (overall survival) of mantle cell lymphoma patients. Categorizations by (**a**) IL2 levels, (**b**) IL17A levels, (**c**) SOX11 positivity, (**d**) Genotypes of *IL2* rs2069762, (**e**) Haplotypes in *IL10* and (**f**) Genotypes of *TGFB1* rs6957. Each graphic contains the hazard ratios and p-values obtained in the respective univariate Cox regressions

Univariate survival analyses of IHC markers showed that the presence of low IL2 levels associated with worse EFS and marginally with OS (*p* = 0.01 and 0.09, respectively) (Fig. 5a and Fig. 6a). High FOXP3/CD3 ratios (*p* = 0.002) and high CD8/CD3 ratios (*p* = 0.03) associated only with worse EFS, and low granzyme B levels presented a trend of association with worse EFS (*p* = 0.06) (Figs. 5b-d). On the other hand, high IL17A levels associated only with shorter OS (*p* = 0.03) (Fig. 6b). SOX11 expression was not associated either with EFS or with OS (Figs. 5e and Fig. 6c).

Regarding the SNVs, presence of the GG genotype of *IL2* rs2069762 negatively affected both EFS (*p* = 0.01, Fig. 5f) and OS (*p* = 0.006, Fig. 6d). In addition, patients carrying the CC haplotype in *IL10*, involving rs3024491 and rs1800872, had improved EFS (*p* = 0.04, Fig. 5g) and OS (*p* = 0.007, Fig. 6e). Besides, the GG genotype of *TGFB1* rs6957, compared with GA + AA genotypes, associated solely with worse OS (*p* = 0.03, Fig. 6f). Finally, the AA+AG genotypes of *TGFBR2* rs3087465 (*p* = 0.001) and the TC + CC genotypes of *IL17F* rs763780 (*p* = 0.03) associated only with worse EFS (Fig. 5h and i).

After multivariate analyses, in the IHC model, high FOXP3/CD3 ratios (HR = 5.03, 95% CI: 1.97–12.84, *p* = 0.001) and low IL2 tumor levels (HR = 2.83, 95% CI: 1.06–7.58, *p* = 0.03) remained independent predictors of worse EFS, whereas high IL17A tumor levels were independently associated with worse OS (HR = 4.68, 95% CI: 1.72–12.77, *p* = 0.003) (Table 2).

**Table 2:** Univariate and multivariate Cox regressions for candidate biomarkers in mantle cell lymphoma survival

Biomarker	Univariate				Multivariate			
	EFS HR (95% CI)	***p***	OS HR (95% CI)	***p***	EFS* HR (95% CI)	***p***	OS* HR (95% CI)	***p***
**Immunohistochemical model**								
**FOXP3/CD3 ratio**								
High	3.16 (1.52–6.58)	**0.002**	1.77 (0.75–4.19)	0.18	5.03 (1.97–12.84)	**0.001**^**a**^	N/A	N/A
Low	Reference		Reference		Reference		Reference	
**CD8/CD3 ratio**								
High	2.22 (1.04–4.77)	**0.03**	1.06 (0.44–2.51)	0.88	1.02 (0.33–3.13)	0.96	N/A	N/A
Low	Reference		Reference		Reference		Reference	
**Granzyme B**								
High	0.50 (0.24–1.04)	0.06	0.69 (0.28–1.67)	0.41	1.18 (0.51–2.72)	0.69	N/A	N/A
Low	Reference		Reference		Reference		Reference	
**IL2**								
High	Reference	**0.01**	Reference		Reference	**0.03**^**b**^	Reference	0.07
Low	2.71 (1.26–5.83)		2.06 (0.88–4.80)	0.09	2.83 (1.06–7.58)		2.50 (0.92–6.77)	
**IL17A**								
High	1.48 (0.73–2.97)	0.27	2.55 (1.06–6.12)	**0.03**	N/A	N/A	4.68 (1.72–12.77)	**0.003**^**c**^
Low	Reference		Reference		Reference		Reference	
**SNV model**								
***IL2*** **rs2069762**								
GG	4.26 (1.56–11.60)	**0.005**	9.05 (2.33–35.05)	**0.001**	3.13 (1.12–8.69)	**0.02**^**d**^	2.73 (0.86–8.67)	0.08
GT+TT	Reference		Reference		Reference		Reference	
***TGFB1*** **rs6957**								
GG	1.86 (0.43–7.92)	0.39	5.38 (1.17–24.73)	**0.03**	N/A	N/A	3.22 (0.57–18.15)	0.18
GA + AA	Reference		Reference		Reference		Reference	
***TGFBR2*** **rs3087465**								
AA+AG	3.40 (1.60–7.22)	**0.001**	1.37 (0.56–3.36)	0.47	4.32 (1.88–9.93)	**0.001**^**e**^	N/A	N/A
GG	Reference		Reference		Reference		Reference	
***IL10*** **CC haplotype**								
Present	0.45 (0.21–0.97)	**0.04**	0.28 (0.11–0.70)	**0.007**	0.32 (0.14–0.74)	**0.008**^**f**^	0.26 (0.10–0.68)	**0.006**^**g**^
Absent	Reference		Reference		Reference		Reference	
***IL17F*** **rs763780**								
TC + CC	0.40 (0.17–0.91)	**0.03**	1.16 (0.34–3.99)	0.80	1.42 (0.47–4.23)	0.52	N/A	N/A
TT	Reference		Reference		Reference		Reference	
**Principal components model**								
Component 3	0.670 (0.40–1.10)	0.12	0.58 (0.31–1.08)	0.08	N/A	N/A	0.77 (0.48–1.24)	0.29
Component 4	1.34 (0.89–2.01)	0.15	1.65 (1.04–2.61)	**0.03**	N/A	N/A	1.68 (1.08–2.62)	**0.02**^**h**^

***EFS*** Event-free survival; ***OS*** Overall survival; ***HR*** Hazard ratio; ***95% CI*** 95% Confidence interval; **SNV** Single nucleotide variant; ***N/A*** Not applicable. **Component 3**: encompasses high levels of NK cells (CD57+), low levels of IL17A and low Ki-67. **Component 4**: encompasses high Ki-67 index and high counts of CD68+ and CD163+ macrophages. (*) Adjustement for B-symptoms and the MIPI index (EFS); adjustement for B-symptoms and bone marrow infiltration (OS).^a^*p* (bootstrap) = 0.02, ^b^*p* (bootstrap) = 0.12, ^c^*p* (bootstrap) = 0.004, ^d^*p* (bootstrap) = 0.12, ^e^*p* (bootstrap) = 0.004, ^f^*p* (bootstrap) = 0.03, ^g^*p* (bootstrap) = 0.11, ^h^*p* (bootstrap) = 0.04

In the multivariate SNV model, presence of the CC haplotype in *IL10* was independently associated both with prolonged EFS (HR = 0.32, 95% CI: 0.14–0.74, *p* = 0.008) and prolonged OS (HR = 0.26, 95% CI: 0.10–0.68, *p* = 0.006). In addition, the GG genotype of *IL2* rs2069762 remained an independent predictor of EFS (HR = 3.13, 95% CI: 1.12–8.69, *p* = 0.02). Finally, the AA+AG genotypes of *TGFBR2* rs3087465 were associated with worse EFS (HR = 4.32, 95% CI:1.88–9.93, *p* = 0.001).

### Principal component analysis

To address the interplay among TME components with a more biologically plausible approach, we used PCA to verify interactions and trends of convergence among the various IHC markers. The final PCA model was based on 6 factors and explained 75.02% of the variance (supplementary Table 9). The first component highlighted the opposition between granzyme B and IL10, IL17A, IL17F and TGFBR1. The second one directly associated FOXP3/CD3 and CD8/CD3 ratios and opposed them against macrophage infiltration (CD68). The third component emphasized the presence of a cytotoxic marker (CD57) that opposed IL17A and the proliferative index Ki-67. On the fourth component, pan-macrophages (CD68+), M2-macrophages (CD163+) and Ki-67 were directly associated. The fifth component aggregated together two cytotoxic markers (granzyme B and perforin), as well as IL2 levels. Finally, the last component inversely associated T-cell levels and IL2.

The presence of the fourth principal component was associated with blastoid cytology, using a linear regression model (*F* = 9.43, *p* = 0.003, *R*^*2*^ = 0.14). No other associations with features at diagnosis were seen.

The third and fourth principal components presented associations with OS in univariate analysis (*p* = 0.08 and 0.03, respectively). After multivariate analysis, only the presence of the fourth component was significantly associated with worse OS (HR = 1.68, 95% CI: 1.08–2.62, *p* = 0.02) (Table 2).

## Discussion

In this cohort of MCL patients, the traditional assessment of TME components in tumor biopsies was complemented with the genotyping of candidate SNVs from immune-response genes. Increased numbers of FOXP3+ lymphocytes, higher IL17A, lower IL2 and a principal component involving Ki67 and macrophages were independently associated with worse outcome in the tumor compartment. Within the genetic counterpart of the TME, SNVs in *IL10*, *TGFBR2* and *IL2* also showed association with prognosis. Our results, although exploratory, provide further evidence that the immune microenvironment poses relevant biological relevance in this disease. This, ultimately, could foster the development of TME-directed therapies that may complement the traditional treatment [1, 13, 50, 51].

The prognostic role of some T-cell subpopulations in MCL was previously assessed by Nygren et al. (2014), who found that the predominance of CD4+ lymphocytes over CD8+ lymphocytes was associated with less aggressive disease [8]. As CD4+ cells represent a heterogeneous group of lymphoid subpopulations, it remains to be elucidated how the balance among different subtypes of CD4+ lymphocytes could explain their findings. We assessed some of these subtypes and found that high FOXP3/CD3 ratios and high IL17A tumor levels were associated with worse outcomes. FOXP3 is traditionally used as a marker of CD4+ regulatory T-cells (T-reg), which were already implicated on immune suppression and worse prognosis of other lymphoma subtypes, such as follicular lymphoma [52]. Complementarily, IL17A is produced by other subtypes of T-CD4+ cells (Th17 cells). It enhances proliferative and pro-angiogenic signals to neoplastic cells, including lymphoma cell lines, which might explain the adverse prognostic role found in our study [53, 54]. Interestingly, the fact that both T-reg cells and IL17A were prognostic in our series also raises the possibility of involvement of “inflammatory T-regs” (IL17 producing T-reg cells) on the immune landscape of MCL [55]. Confirmation of this hypothesis relies on additional studies performing experiments such as double immunostainings.

When evaluating the TME by PCA, tumors rich in CD163 and with a high proliferative index had a worse prognosis. An association with the blastoid cytomorphology was also observed. The presence of higher Ki-67 is a known prognostic factor in MCL [2], and a higher density of CD163+ macrophages was previously associated with adverse clinical features in this disease [7]. Recently, an interplay between M2-macrophages and MCL cells was also described as relevant for the production of CSF1 and survival of lymphoma cells [13], which might explain the adverse prognostic role of the PCA detected in the present study.

Regarding the genetic counterpart of the TME, we found that the CC haplotype in *IL10* (composed of the “C” alleles of rs3024491 and rs1800872) was a predictor of better OS and EFS in MCL. In previous reports addressing patients with Hodgkin’s lymphoma and B-cell lymphomas, similar results were achieved for the second SNV individually [56, 57]. It was already suggested that the locus involving rs1800872 might modulate IL10 production, however, the results concerning this modulation are conflicting [56]. On the other hand, Assis et al. [29] reported that the “A” allele of rs3024491 associated with higher production of IL10 than the “C” allele, a finding that might have an impact on the CC haplotype. In our samples, we did not find any evidence of changes in local IL10 production either by the SNVs or by the CC haplotype; however, the systemic levels of this interleukin need to be addressed in further studies. A reduction of IL10 production by the haplotype seems a plausible explanation for the prolonged survival of CC patients, because IL10 induces a pro-tumorigenic microenvironment in MCL [13].

Another potentially targetable pathway in MCL is TGFβ and related proteins. Rizzatti et al. (2005) described that several genes from the *TGFB* superfamily were up-regulated in MCL samples, compared to controls, but this was little explored in further studies [32]. In our cohort, we found that the AA+AG genotypes of *TGFBR2* rs3087465 independently associated with poorer EFS. To the best of our knowledge, this is the first association of this SNV with survival in cancer patients. The” A” allele of rs3087465 was previously found to increase the promoter activity of TGFBR2 [37], but its specific role in MCL and the mechanisms influencing survival in our cohort are yet to be elucidated. In the same pathway, the SNV *TGFB1* rs6957 was not a predictor of outcome after multivariate analysis; however, the GG genotype was associated with decreased levels of TGFβ and CD163+ macrophages in MCL biopsies. These findings are similar to a previous report in asthma patients, in whom this SNV also modulated macrophage proliferation [33]. However, the low frequency of the GG genotype of rs6957 demands further investigation in larger sample sizes.

Finally, considering the cytotoxic (Th1) immune-response pathway, we found that the GG genotype of *IL2* rs2069762 was an independent predictor of worse EFS. This mirrors the findings of Cerhan et al. (2007), in which the same genotype worsened the survival of follicular lymphoma patients in the pre-rituximab era [15]. Interestingly, the GG genotype was previously associated with reduction of IL2 production in healthy individuals [58]. Even though the SNV was not associated with modulation of IL2 levels in our samples, the presence of low scores of IL2, in the IHC model, was associated with worse EFS. Taken together, these results endorse the role of IL2-dependent cytotoxicity observed in MCL experimental models [26]. Therefore, further investigation of this pathway in patient-derived samples should be fostered.

In our cohort of MCL, SOX11 expression was not associated with prognosis. The importance of this transcription factor for the diagnosis of MCL is already established [59]. More recently, the role of this molecule in MCL homing and migration was also proposed [60]. However, a possible prognostic role remains in debate. This is illustrated by controversial results associating SOX11 negativity with both better and worse clinical outcomes in this disease [61, 62]. We believe that this controversy might be due to different approaches (gene or protein expression), and also due to the lack of a standardized cut-off for the immunohistochemical expression of SOX11 [63].

The limited sample size represents a drawback of this study. Furthermore, the two institutions involved in our study present different sociodemographical profiles that might influence response to therapy [64]. In addition, we were unable to assess *TP53* mutational status, which is a well-known prognostic factor in MCL [65]. Therefore, we recognize the need of independent studies to validate our findings. However, the genotyping of SNVs in immune response genes, as well as the use of PCA in TME subpopulations, enabled a novel and more integrative approach to address the TME in MCL. Also, our survival analyses were performed only in patients that received anti-CD20; hence, our findings present translational relevance. Finally, the majority of the IHC markers were analyzed quantitatively and automatically, minimizing the risk of bias and poor reproducibility of manual scoring in lymphoma studies [66].

## Conclusions

This is the first study to provide a broader approach of the TME in MCL, by evaluating both the TME immune cell composition in biopsies and SNVs within immune-response genes. Our study supports the associations of tumor FOXP3/CD3 ratios, IL17A and IL2 with outcome in the rituximab era. We also demonstrate, in the same cohort, the prognostic roles of *TGFBR2* rs3087465, *IL2* rs2069762 and the CC haplotype of *IL10*. The distinct approach presented herein might contribute to novel insights in the biology of MCL, and in future studies considering new therapeutic options in this lymphoma.

## Supplementary Information

**Additional file 1: Supplementary Table 1.** Primary antibodies used for immunohistochemistry in this study.

**Additional file 2: Supplementary Table 2.** Clinicopathological features of the mantle cell lymphoma patients in this study.

**Additional file 3: Supplementary Table 3.** Cellular microenvironment composition and clinicopathological features of mantle cell lymphoma.

**Additional file 4: Supplementary Table 4.** Intratumoral expression of cytokines and clinicopathological features of mantle cell lymphoma.

**Additional file 5: Supplementary Table 5.** SOX11 expression and clinicopathological features in mantle cell lymphoma.

**Additional file 6: Supplementary Table 6.** Hardy-Weinberg equilibrium testing for all the SNVs assessed in this study.

**Additional file 7: Supplementary Table 7.** Frequencies of haplotypes in *IL10* and *IL12A* in mantle cell lymphoma patients.

**Additional file 8: Supplementary Table 8.** Univariate Cox regression for clinicopathological features influencing survival of mantle cell lymphoma patients.

**Additional file 9: Supplementary Table 9**. Principal component analysis of the immunohistochemical variables in mantle cell lymphoma.

**Additional file 10: Supplementary Fig. 1**. Representative photomicrographs of tumor-infiltrating lymphocytes in mantle cell lymphoma. **(A)** CD3**, (B)** CD4**, (C)** CD8**, (D)** CD57**, (E)** FOXP3**, (F)** PD1**, (G)** Granzyme **B, (H)** Perforin. Each letter is sub-labeled as “1”, “2” and “3”, representing, respectively, cases with weak, intermediate and strong positivity. All images were obtained at a 200x magnification.

**Additional file 11: Supplementary Fig. 2**. Representative photomicrographs of macrophages, iNOS staining and the proliferative index in mantle cell lymphoma. **(A)** CD68**, (B)** CD163**, (C)** iNOS**, (D)** Ki67. Each letter is sub-labeled as “1”, “2” and “3”, representing, respectively, cases with weak, intermediate and strong positivity. All images were obtained at a 200x magnification.

**Additional file 12: Supplementary Fig. 3**. Representative photomicrographs of cytokines’ stainings in mantle cell lymphoma. **(A)** IL12A**, (B)** IL2**, (C)** IL10**, (D)** TGFβ**, (E)** TGFBR1**, (F)** TGFBR2**, (G)** IL17A**, (H)** IL17F. Each letter is sub-labeled as “1″, “2″ and “3″, representing, respectively, cases with weak, intermediate and strong positivity. The exception is for TGFBR2, in which no convincing positivity was observed; therefore, F1, F2 and F3 represent negative cases. All images were obtained at a 200x magnification.

**Additional file 13: Supplementary Fig. 4.** Representative photomicrographs of SOX11 assessment in mantle cell lymphoma. **(A)**SOX11^high^ case. **(B)**SOX11^low^ case.

**Additional file 14: Supplementary Fig. 5.** Tumor microenvironment markers (**A-R**) and proliferation index (**S**) in SOX11^high^ and SOX11^low^ mantle cell lymphoma cases. Horizontal lines represent the median levels, and the whiskers show interquartile ranges.

**Additional file 15: Supplementary Fig. 6.** Linkage disequilibrium (LD) plots in mantle cell lymphoma patients for **(A)** *IL12A* and **(B)** *IL10* genes. In each square, the LD is measured between groups of single nucleotide variants. Higher values of LD (expressed as D′) are shown in red squares.

## Data Availability

The datasets used during the current study are available from the corresponding author on reasonable request.

## Abbreviations

95%CI: 95% Confidence interval
D’: Linkage disequilibrium coefficient
EFS: Event-free survival
FFPE: Formalin-fixed, paraffin embedded
HWE: Hardy-weinberg equilibrium
IHC: Immunohistochemical
LD: Linkage disequilibrium
MCL: Mantle cell lymphoma
MIPI: Mantle cell lymphoma international prognostic index
NHL: Non-hodgkin lymphoma
OR: Odds ratio
OS: Overall survival
PCA: Principal component analysis
SNVs: Single nucleotide variants
TMA: Tissue microarray
TME: Tumor microenvironment
WHO: World health Organization
