# Supplementary material for: Single nucleotide variants in immune-response genes and the tumor microenvironment composition predict progression of mantle cell lymphoma
Source: BMC Cancer. 2021 Mar 1;21:209. doi: 10.1186/s12885-021-07891-9 (PMC7919095; doi:10.1186/s12885-021-07891-9)
Supplement: Supplementary file 1 — Additional file 1: Supplementary Table 1. Primary antibodies used for immunohistochemistry in this study. [file 12885_2021_7891_MOESM1_ESM.docx]

| **Supplementary table 1.** Primary antibodies used for immunohistochemistry in this study. | | | |
| --- | --- | --- | --- |
| **Marker** | **Dilution** | **Supplier** | **Supplier code** |
| **CD3** | 1:100 | BioSB | BSB5146 |
| **CD4** | 1:50 | BioSB | BSB5153 |
| **FOXP3** | 1:25 | BioSB | BSB6763 |
| **PD1** | 1:100 | Abcam | ab137132 |
| **CD8** | 1:500 | BioSB | BSB5174 |
| **Granzyme B** | 1:400 | BioSB | BSB5594 |
| **Perforin** | 1:10 | BioSB | BSB2110 |
| **CD57** | Pure | BioSB | BSB10 |
| **CD68** | 1:500 | BioSB | BSB5293 |
| **CD163** | 1:100 | BioSB | BSB6308 |
| **IL2** | 1:800 | Abcam | ab92381 |
| **IL10** | 1:150 | Abcam | ab34843 |
| **IL12A** | 1:10000 | Abcam | ab131039 |
| **IL17A** | 1:50 | Abcam | ab136668 |
| **IL17F** | 1:25 | Abcam | ab168194 |
| **TGFβ** | 1:300 | Abcam | ab27969 |
| **TGFBR1** | 1:50 | Abcam | ab31013 |
| **TGFBR2** | 1:25 | Abcam | ab61213 |
| **iNOS** | 1:8000 | Abcam | ab129372 |
| **SOX11** | 1:100 | Cell Marque | 382M-14 |
| **Ki67** | 1:150 | Dako | M7240 |
