# Supplementary material for: Single nucleotide variants in immune-response genes and the tumor microenvironment composition predict progression of mantle cell lymphoma
Source: BMC Cancer. 2021 Mar 1;21:209. doi: 10.1186/s12885-021-07891-9 (PMC7919095; doi:10.1186/s12885-021-07891-9)
Supplement: Supplementary file 2 — Additional file 2: Supplementary Table 2. Clinicopathological features of the mantle cell lymphoma patients in this study. [file 12885_2021_7891_MOESM2_ESM.docx]

| **Supplementary table 2.** Clinicopathological features of the mantle cell lymphoma patients in this study. | |
| --- | --- |
| **Characteristic** | **Number of patients (%)** |
| **B symptoms** |  |
| Present (%) | 60 (49.2) |
| Absent (%) | 57 (46.7) |
| Not available (%) | 5 (4.1) |
| **Bulky disease** |  |
| Present (%) | 21 (17.2) |
| Absent (%) | 83 (68.0) |
| Not available (%) | 18 (14.8) |
| **Bone marrow infiltration** |  |
| Present (%) | 74 (60.6) |
| Absent (%) | 41 (33.6) |
| Not available (%) | 7 (5.8) |
| **Extranodal disease, excluding bone marrow** |  |
| Present (%) | 47 (38.5) |
| Absent (%) | 65 (53.3) |
| Not available (%) | 10 (8.2) |
| **Ann Arbor stage** |  |
| I or II (%) | 15 (12.3) |
| III ou IV (%) | 107 (87.7) |
| **MIPI** |  |
| High risk (%) | 43 (35.3) |
| Intermediate risk (%) | 33 (27.0) |
| Low risk (%) | 30 (24.6) |
| Not available (%) | 16 (13.1) |
| **Cytologic pattern** |  |
| Classic (%) | 76 (62.3) |
| Small cell (%) | 15 (12.3) |
| Blastoid (%) | 11 (9.0) |
| Not available (%) | 20 (16.4) |
| **Architectural pattern** |  |
| Diffuse (%) | 70 (57.4) |
| Nodular (%) | 15 (12.3) |
| Mantle zone (%) | 2 (1.6) |
| Not available (%) | 35 (28.7) |
| **Characteristic** | **Number of patients (%)** |
| **First-line treatment** |  |
| R-CHOP/R-CHOP like (%) | 68 (55.7) |
| R-CVP (%) | 6 (4.9) |
| R-HyperCVAD (%) | 3 (2.5) |
| Rituximab monotherapy (%) | 1 (0.8) |
| CHOP/CHOP like (%) | 22 (18.0) |
| Other drugs (%) | 18 (14.8) |
| Watch and wait (%) | 4(3.3) |

MIPI=Mantle Cell Lymphoma International Prognostic Index.
