# Supplementary material for: Single nucleotide variants in immune-response genes and the tumor microenvironment composition predict progression of mantle cell lymphoma
Source: BMC Cancer. 2021 Mar 1;21:209. doi: 10.1186/s12885-021-07891-9 (PMC7919095; doi:10.1186/s12885-021-07891-9)
Supplement: Supplementary file 3 — Additional file 3: Supplementary Table 3. Cellular microenvironment composition and clinicopathological features of mantle cell lymphoma. [file 12885_2021_7891_MOESM3_ESM.docx]

|  | **Supplementary table 3.** Cellular microenvironment composition and clinicopathological features of mantle cell lymphoma. | | | | | | | | | | | | | | |
| --- | --- | --- | --- | --- | --- | --- | --- | --- | --- | --- | --- | --- | --- | --- | --- |
|  | | **CD3 high** | **CD4 high** | **CD8 high** | **CD4/CD3 high** | **CD8/CD3**  **high** | **CD4/CD8 high** | **Granzime B**  **high** | **Perforin high** | **PD1**  **high** | **FOXP3**  **high** | **FOXP3/CD3**  **high** | **CD68**  **high** | **CD163**  **high** |  |
| **B-symptoms** | |  |  |  |  |  |  |  |  |  |  |  |  |  |  |
| Present (%) | | 22/47 (46.8) | 24/43 (55.8) | 19/41 (46.3) | 24/42 (57.1) | 23/38 (60.5) | 18/39 (46.1) | 25/45 (55.5) | 23/42 (54.7) | 14/39 (35.8) | 39/44 (88.6) | 22/44 (50.0) | 17/45 (37.7) | 26/46 (56.5) |  |
| Absent (%) | | 20/39 (51.2) | 17/38 (44.7) | 20/38 (52.6) | 17/37 (45.9) | 15/36 (41.6) | 21/37 (56.7) | 15/37 (40.5) | 17/37 (45.9) | 23/36 (63.8) | 22/39 (56.4) | 19/37 (51.3) | 23/38 (60.5) | 16/38 (42.1) |  |
| P-value* | | 0.67 | 0.32 | 0.57 | 0.32 | 0.10 | 0.85 | 0.17 | 0.43 | **0.04*** | 0.22 | 0.90 | 0.12 | 0.18 |  |
| **MIPI risk** | |  |  |  |  |  |  |  |  |  |  |  |  |  |  |
| High (%) | | 9/28 (32.1) | 10/26 (38.4) | 13/25 (52.0) | 10/24 (41.6) | 17/23 (73.9) | 8/24 (33.3) | 13/27 (48.1) | 15/26 (57.6) | 13/26 (50.0) | 15/28 (53.5) | 17/27 (62.9) | 9/28 (32.1) | 14/29 (48.2) |  |
| Low/intermediate (%) | | 30/48 (62.5) | 28/46 (60.8) | 25/48 (52.0) | 24/46 (52.1) | 17/45 (37.7) | 29/46 (63.0) | 20/45 (44.4) | 20/44 (45.4) | 22/41 (53.6) | 23/45 (51.1) | 18/45 (40.0) | 29/45 (64.4) | 25/45 (55.5) |  |
| P-value* | | **0.04*** | 0.06 | 0.99 | 0.40 | **0.02*** | **0.04*** | 0.76 | 0.32 | 0.77 | 0.83 | 0.05 | **0.02*** | 0.54 |  |
| **Bone marrow infiltration** | |  |  |  |  |  |  |  |  |  |  |  |  |  |  |
| Present (%) | | 21/44 (47.7) | 22/42 (52.3) | 20/41 (48.7) | 22/41 (53.6) | 21/37 (56.7) | 20/40 (50.0) | 20/46 (43.4) | 18/41 (43.9) | 20/40 (50.0) | 22/43 (51.1) | 24/41 (58.5) | 23/43 (53.4) | 26/44 (59.1) |  |
| Absent (%) | | 19/37 (51.3) | 15/34 (44.1) | 17/32 (53.1) | 15/33 (45.4) | 15/31 (48.3) | 15/30 (50.0) | 18/34 (52.9) | 20/33 (60.6) | 15/29 (51.7) | 19/35 (54.2) | 17/34 (50.0) | 15/35 (42.8) | 15/35 (42.8) |  |
| P-value* | | 0.74 | 0.47 | 0.71 | 0.48 | 0.49 | 1.00 | 0.57 | 0.15 | 0.88 | 0.78 | 0.53 | 0.35 | 0.15 |  |
| **Cytology** | |  |  |  |  |  |  |  |  |  |  |  |  |  |  |
| Blastoid (%) | | 3/9 (33.3) | 3/9 (33.3) | 3/8 (37.5) | 6/9 (66.6) | 3/8 (37.5) | 5/8 (62.5) | 5/9 (55.5) | 7/9 (77.7) | 5/7 (71.4) | 3/9 (33.3) | 6/9 (66.6) | 6/9 (66.6) | 6/9 (66.6) |  |
| Non-blastoid (%) | | 39/75 (52.0) | 38/71 (53.5) | 36/69 (52.1) | 32/69 (46.3) | 34/66 (51.5) | 31/66 (47.0) | 36/72 (50.0) | 34/69 (49.2) | 33/66 (50.0) | 39/73 (53.4) | 34/71 (47.8) | 23/73 (31.5) | 37/74 (50.0) |  |
| P-value* | | 0.29 | 0.25 | 0.43 | 0.25 | 0.45 | 0.47 | 0.75 | 0.10 | 0.28 | 0.30 | 0.28 | 0.29 | 0.48 |  |

(*)After adjustement for multiple comparisons (Benjamini-Hochberg method). “High” values refer to values above the median levels. P-values were obtained from chi-squared tests.
