# Supplementary material for: Single nucleotide variants in immune-response genes and the tumor microenvironment composition predict progression of mantle cell lymphoma
Source: BMC Cancer. 2021 Mar 1;21:209. doi: 10.1186/s12885-021-07891-9 (PMC7919095; doi:10.1186/s12885-021-07891-9)
Supplement: Supplementary file 4 — Additional file 4: Supplementary Table 4. Intratumoral expression of cytokines and clinicopathological features of mantle cell lymphoma. [file 12885_2021_7891_MOESM4_ESM.docx]

| **Supplementary table 4.** Intratumoral expression of cytokines and clinicopathological features of mantle cell lymphoma. | | | | | | | |
| --- | --- | --- | --- | --- | --- | --- | --- |
|  | **IL12A**  **high** | **IL2**  **high** | **IL10 high** | **TGFβ**  **high** | **TGFBR1 high** | **IL17A**  **high** | **IL17F**  **high** |
| **B-symptoms** |  |  |  |  |  |  |  |
| Present (%) | 23/46 (50.0) | 20/45 (44.4) | 21/45 (46.6) | 22/44 (50.0) | 21/41 (51.2) | 21/45 (46.6) | 19/45 (42.2) |
| Absent (%) | 21/39 (53.8) | 21/38 (55.2) | 22/38 (57.8) | 20/37 (54.0) | 19/36 (52.7) | 21/38 (55.2) | 24/38 (63.1) |
| P-value* | 0.72 | 0.32 | 0.30 | 0.71 | 0.89 | 0.43 | 0.05 |
| **MIPI** |  |  |  |  |  |  |  |
| High risk (%) | 16/29 (55.1) | 16/29 (55.1) | 16/28 (57.1) | 15/26 (57.6) | 9/25 (36.0) | 10/27 (37.0) | 11/29 (37.9) |
| Low/intermediate risks (%) | 25/46 (54.3) | 20/45 (44.4) | 25/45 (55.5) | 25/45 (55.5) | 25/43 (58.1) | 26/46 (56.5) | 27/44 (61.3) |
| P-value* | 0.94 | 0.36 | 0.89 | 0.86 | 0.07 | 0.10 | 0.05 |
| **Bone marrow infiltration** |  |  |  |  |  |  |  |
| Present (%) | 29/44 (65.9) | 19/42 (45.2) | 23/43 (53.4) | 22/43 (51.1) | 22/40 (55.0) | 22/43 (51.1) | 25/43 (58.1) |
| Absent (%) | 12/36 (33.3) | 19/36 (52.7) | 16/35 (45.7) | 16/33 (48.4) | 14/32 (43.7) | 17/35 (48.5) | 16/35 (45.7) |
| P-value* | **0.01*** | 0.50 | 0.49 | 0.81 | 0.34 | 0.82 | 0.27 |
| **Cytology** |  |  |  |  |  |  |  |
| Blastoid (%) | 4/9 (44.4) | 3/9 (33.3) | 4/9 (44.4) | 4/9 (44.4) | 6/9 (66.6) | 8/9 (88.8) | 5/9 (55.5) |
| Non-blastoid (%) | 37/75 (49.3) | 38/73 (52.0) | 16/73 (21.9) | 36/71 (50.7) | 33/67 (49.2) | 33/73 (45.2) | 36/73 (49.3) |
| P-value* | 1.00 | 0.48 | 1.00 | 1.00 | 0.48 | **0.04*** | 0.72 |

(*)After adjustement for multiple comparisons (Benjamini-Hochberg method). “High” values refer to values above the median levels. P-values were obtained from chi-squared tests.
