# Supplementary material for: Single nucleotide variants in immune-response genes and the tumor microenvironment composition predict progression of mantle cell lymphoma
Source: BMC Cancer. 2021 Mar 1;21:209. doi: 10.1186/s12885-021-07891-9 (PMC7919095; doi:10.1186/s12885-021-07891-9)
Supplement: Supplementary file 5 — Additional file 5: Supplementary Table 5. SOX11 expression and clinicopathological features in mantle cell lymphoma. [file 12885_2021_7891_MOESM5_ESM.docx]

| **Supplementary table 5.** SOX11 expression and clinicopathological features in mantle cell lymphoma. | | | |
| --- | --- | --- | --- |
| **Characteristic** | **SOX11^high^**  **(N=76)** | **SOX11^low^**  **(N=13)** | **P-value** |
| **Median age (range)** | 66.5 (33-93) | 66 (46-76) | 0.56^a^ |
| **Gender** |  |  |  |
| Male (%) | 61 (80.3) | 7 (53.8) | **0.03^b^** |
| Female (%) | 15 (19.7) | 6 (46.2) |  |
| **B symptoms** |  |  |  |
| Present (%) | 43 (56.6) | 4 (30.8) | 0.33^c^ |
| Absent (%) | 33 (43.4) | 7 (53.8) |  |
| Not available (%) | 0 (0.0) | 2 (15.4) |  |
| **Bulky disease** |  |  |  |
| Present (%) | 11 (14.5) | 4 (30.8) | 0.22 ^c^ |
| Absent (%) | 51 (67.1) | 7 (53.8) |  |
| Not available (%) | 14 (18.4) | 2 (15.4) |  |
| **Bone marrow infiltration** |  |  |  |
| Present (%) | 43 (56.6) | 5 (38.5) | 0.22 ^c^ |
| Absent (%) | 27 (35.5) | 7 (53.8) |  |
| Not available (%) | 6 (7.9) | 1 (7.7) |  |
| **Extranodal disease, excluding bone marrow** |  |  |  |
| Present (%) | 32 (42.1) | 5 (38.5) | 1.00^c^ |
| Absent (%) | 39 (51.3) | 7 (53.8) |  |
| Not available (%) | 5 (6.6) | 1 (7.7) |  |
| **Ann Arbor stage** |  |  |  |
| I or II (%) | 10 (13.2) | 0 (0.0) | 0.34 ^c^ |
| III ou IV (%) | 66 (86.8) | 13 (100.0) |  |
| **MIPI** |  |  |  |
| High risk (%) | 27 (35.5) | 3 (23.1) | 0.73^d^ |
| Intermediate risk (%) | 20 (26.4) | 5 (38.4) |  |
| Low risk (%) | 21 (27.6) | 2 (15.4) |  |
| Not available (%) | 8 (10.5) | 3 (23.1) |  |
| **Characteristic** | **SOX11^high^**  **(N=76)** | **SOX11^low^**  **(N=13)** | **P-value** |
| **Cytologic pattern** |  |  |  |
| Classic (%) | 57 (75.0) | 9 (69.2) | 1.00^e^ |
| Small cell (%) | 7 (9.2) | 3 (23.1) |  |
| Blastoid (%) | 9 (11.8) | 1 (7.7) |  |
| Not available (%) | 3 (4.0) | 0 (0.0) |  |
| **Architectural pattern** |  |  |  |
| Diffuse (%) | 52 (68.4) | 11 (84.6) | 1.00^f^ |
| Nodular (%) | 12 (15.8) | 2 (15.4) |  |
| Mantle zone (%) | 2 (2.6) | 0 (0.0) |  |
| Not available (%) | 10 (13.2) | 0 (0.0) |  |
| **First-line treatment** |  |  |  |
| R-CHOP/R-CHOP like (%) | 44 (57.9) | 7 (53.8) |  |
| R-CVP (%) | 2 (2.6) | 0 (0.0) |  |
| R-HyperCVAD (%) | 1 (1.3) | 1 (7.7) | 1.00^g^ |
| Rituximab monotherapy (%) | 0 (0.0) | 0 (0.0) |  |
| CHOP/CHOP like (%) | 15 (19.7) | 1 (7.7) |  |
| Other drugs (%) | 11 (14.5) | 4 (30.8) |  |
| Watch and wait (%) | 3 (4.0) | 0 (0.0) |  |

MIPI=Mantle Cell Lymphoma International Prognostic Index. ^a^Mann-Whitney test, ^b^Chi-squared test, ^c^Fisher’s exact test, ^d^Fisher’s exact test (high-risk vs others), ^e^Fisher’s exact test (blastoid cytology vs others), ^f^Fisher’s exact test (diffuse pattern vs others), ^g^ Fisher’s exact test (regimens containing rituximab vs others).
