# Supplementary material for: Single nucleotide variants in immune-response genes and the tumor microenvironment composition predict progression of mantle cell lymphoma
Source: BMC Cancer. 2021 Mar 1;21:209. doi: 10.1186/s12885-021-07891-9 (PMC7919095; doi:10.1186/s12885-021-07891-9)
Supplement: Supplementary file 6 — Additional file 6: Supplementary Table 6. Hardy-Weinberg equilibrium testing for all the SNVs assessed in this study. [file 12885_2021_7891_MOESM6_ESM.docx]

| **Supplementary table 6.** Hardy-Weinberg equilibrium testing for all the SNVs assessed in this study. | |
| --- | --- |
| **Gene and SNV** | **Findings in mantle cell lymphoma patients** |
| ***IL12A* rs568408** | n=94; *χ^2^=* 0.09  p=0.76 |
| ***IL12A* rs755004** | n=95; *χ^2^=* 0.80  p=0.37 |
| ***IL12A* rs485497** | n=95; *χ^2^=* 0.25  p=0.61 |
| ***IL12A* rs583911** | n=94; *χ^2^=* 0.17  p=0.67 |
| ***IL2* rs2069762** | n=94; *χ^2^=* 0.22  p=0.63 |
| ***IL2* rs6822844** | n=95; *χ^2^=* 0.52  p=0.46 |
| ***IL10* rs1800872** | n=95; *χ^2^=* 0.002  p=0.95 |
| ***IL10* rs3024491** | n=95; *χ^2^=* 0.005  p=0.93 |
| ***IL10* rs1800890** | n=95; *χ^2^=* 0.88  p=0.34 |
| ***TGFB1* rs1800469** | n=94; *χ^2^=* 1.11  p=0.29 |
| ***TGFB1* rs1800471** | n=95; *χ^2^=* 0.52  p=0.46 |
| ***TGFB1* rs6957** | n=95; *χ^2^=* 0.01  p=0.89 |
| ***TGFBR1* rs334348** | n=91; *χ^2^=* 10.9  **p<0.01** |
| ***TGFBR2* rs3087465** | n=95; *χ^2^=* 2.06  p=0.15 |
| ***IL17A* rs3748067** | n=90; *χ^2^=*1.56  p=0.21 |
| ***IL17F* rs763780** | n=92; *χ^2^=*0.99  p=0.31 |
