# Supplementary material for: Single nucleotide variants in immune-response genes and the tumor microenvironment composition predict progression of mantle cell lymphoma
Source: BMC Cancer. 2021 Mar 1;21:209. doi: 10.1186/s12885-021-07891-9 (PMC7919095; doi:10.1186/s12885-021-07891-9)
Supplement: Supplementary file 7 — Additional file 7: Supplementary Table 7. Frequencies of haplotypes in IL10 and IL12A in mantle cell lymphoma patients. [file 12885_2021_7891_MOESM7_ESM.docx]

| **Supplementary table 7.** Frequencies of haplotypes in *IL10* and *IL12A* in mantle cell lymphoma patients. | |
| --- | --- |
| **Gene and haplotype** | **Estimated frequency** |
| ***IL10* CCT** | 74 / 95 = 77.8% |
| ***IL10* ACT** | 48 / 95 = 50.5% |
| ***IL10* CAA** | 12 / 95 = 12.6% |
| ***IL10* CC^a^** | 75 / 95 = 78.9% |
| ***IL10* AC^a^** | 50 / 95 = 52.6% |
| ***IL10* CA^b^** | 53 / 95 = 55.7% |
| ***IL10* CT^b^** | 82 / 95 = 86.3% |
| ***IL10* AA^b^** | 12 / 95 = 12.6% |
| ***IL12A* GGA** | 60 / 95 = 63.1% |
| ***IL12A* AGG** | 64 / 95 = 67.3% |
| ***IL12A* AAG** | 26 / 95 = 27.3% |
| ***IL12A* GGG** | 62 / 95 = 65.2% |
| ***IL12A* GAG** | 74 / 95 = 77.8% |
| ***IL12A* GAA** | 28 / 95 = 29.4% |
| ***IL12A* GG^c^** | 62 / 95 = 65.2% |
| ***IL12A* AG^c^** | 74 / 95 = 77.8% |
| ***IL12A* AA^c^** | 28 / 95 = 29.4% |
| ***IL12A* GA^d^** | 70 / 95 = 73.6% |
| ***IL12A* GG^d^** | 67 / 95 = 70.5% |
| ***IL12A* AG^d^** | 26 / 95 = 27.3% |

**(a).** Haplotype involving rs3024491 e rs1800872.

**(b).** Haplotype involving rs1800872 e rs1800890.

**(c).** Haplotype involving rs583911 e rs568408.

**(d)** Haplotype involving rs568408 e rs485497.
