# Supplementary material for: Single nucleotide variants in immune-response genes and the tumor microenvironment composition predict progression of mantle cell lymphoma
Source: BMC Cancer. 2021 Mar 1;21:209. doi: 10.1186/s12885-021-07891-9 (PMC7919095; doi:10.1186/s12885-021-07891-9)
Supplement: Supplementary file 8 — Additional file 8: Supplementary Table 8. Univariate Cox regression for clinicopathological features influencing survival of mantle cell lymphoma patients. [file 12885_2021_7891_MOESM8_ESM.docx]

| **Supplementary table 8.** Univariate Cox regression for clinicopathological features influencing survival of mantle cell lymphoma patients. | | | | |  |
| --- | --- | --- | --- | --- | --- |
|  | **EFS**  **HR (95% CI)** | ***p*** | **OS**  **HR (95% CI)** | ***p*** | |
| **MIPI** |  |  |  |  | |
| High risk | 1.70 (0.93-3.10) | 0.08 | 1.16 (0.55-2.42) | 0.68 | |
| Low/intermediate risk | Reference |  | Reference |  | |
| **B symptoms** |  |  |  |  | |
| Yes | 2.17 (1.19-3.96) | **0.01** | 2.79 (1.31-5.94) | **0.007** | |
| No | Reference |  | Reference |  | |
| **Bone marrow infiltration** |  |  |  |  | |
| Yes | 1.67 (0.88-3.17) | 0.11 | 2.42 (1.03-5.66) | **0.04** | |
| No | Reference |  | Reference |  | |

**EFS** = event-free survival; **OS** = overall survival; **HR** = hazard ratio; **95% CI** = 95% confidence interval; **MIPI**: Mantle Cell Lymphoma International Prognostic Index.
