# Supplementary material for: Single nucleotide variants in immune-response genes and the tumor microenvironment composition predict progression of mantle cell lymphoma
Source: BMC Cancer. 2021 Mar 1;21:209. doi: 10.1186/s12885-021-07891-9 (PMC7919095; doi:10.1186/s12885-021-07891-9)
Supplement: Supplementary file 9 — Additional file 9: Supplementary Table 9. Principal component analysis of the immunohistochemical variables in mantle cell lymphoma. [file 12885_2021_7891_MOESM9_ESM.docx]

| **Supplementary table 9**. Principal component analysis of the immunohistochemical variables in mantle cell lymphoma. | | | | | | |
| --- | --- | --- | --- | --- | --- | --- |
|  | **Component number** | | | | | |
|  | **1** | **2** | **3** | **4** | **5** | **6** |
| **PD1** |  |  | 0.576 | 0.308 |  |  |
| **IL17A** | 0.344 |  | -0.684 |  |  |  |
| **IL2** |  | 0.629 |  |  | 0.524 | -0.370 |
| **IL10** | 0.772 |  |  |  |  |  |
| **Granzyme B** | -0.526 |  |  |  | 0.657 |  |
| **CD68** | 0.528 | -0.443 |  | 0.511 |  |  |
| **CD163** |  |  | 0.311 | 0.751 |  |  |
| **TGFBR1** | 0.862 |  |  |  |  |  |
| **CD4/CD3 ratio** |  |  |  |  |  | 0.884 |
| **KI67** |  |  | -0.340 | 0.741 |  |  |
| **CD8/CD3 ratio** |  | 0.779 |  |  |  | 0.334 |
| **Perforin** | 0.387 |  |  |  | 0.693 |  |
| **FOXP3/CD3 ratio** |  | 0.882 |  |  |  |  |
| **IL17F** | 0.864 |  |  |  |  |  |
| **CD57** |  |  | 0.78 |  |  |  |
| **Individual variance** | 19.99% | 14.69% | 12.04% | 10.77% | 8.93% | 8.60% |

**Note**: The numbers inside boxes represent the eigenvectors within a component. Reddish tones indicate lower eigenvector values, and blueish tones, higher ones (varying from -1.0 to +1.0). Considering a single component, similar colors point towards direct relationships, whereas opposite colors indicate inverse relationships.
