# Supplementary figures and images for: Single nucleotide variants in immune-response genes and the tumor microenvironment composition predict progression of mantle cell lymphoma
Source: BMC Cancer. 2021 Mar 1;21:209. doi: 10.1186/s12885-021-07891-9 (PMC7919095; doi:10.1186/s12885-021-07891-9)

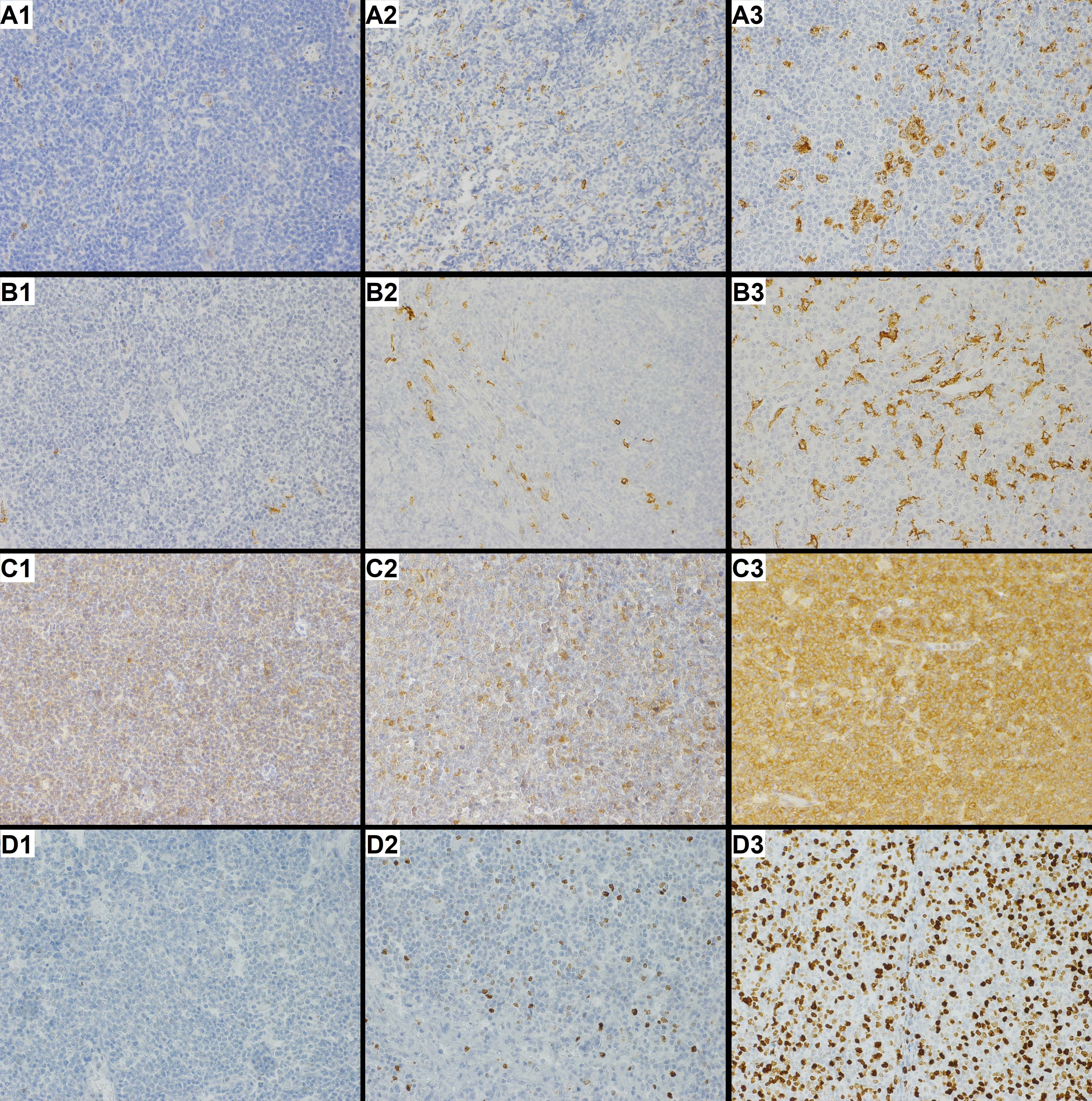

Supplement: Supplementary file 11 — Additional file 11: Supplementary Fig. 2. Representative photomicrographs of macrophages, iNOS staining and the proliferative index in mantle cell lymphoma. (A) CD68, (B) CD163, (C) iNOS, (D) Ki67. Each letter is sub-labeled as “1”, “2” and “3”, representing, respectively, cases with weak, intermediate and strong positivity. All images were obtained at a 200x magnification. [file 12885_2021_7891_MOESM11_ESM.tif]

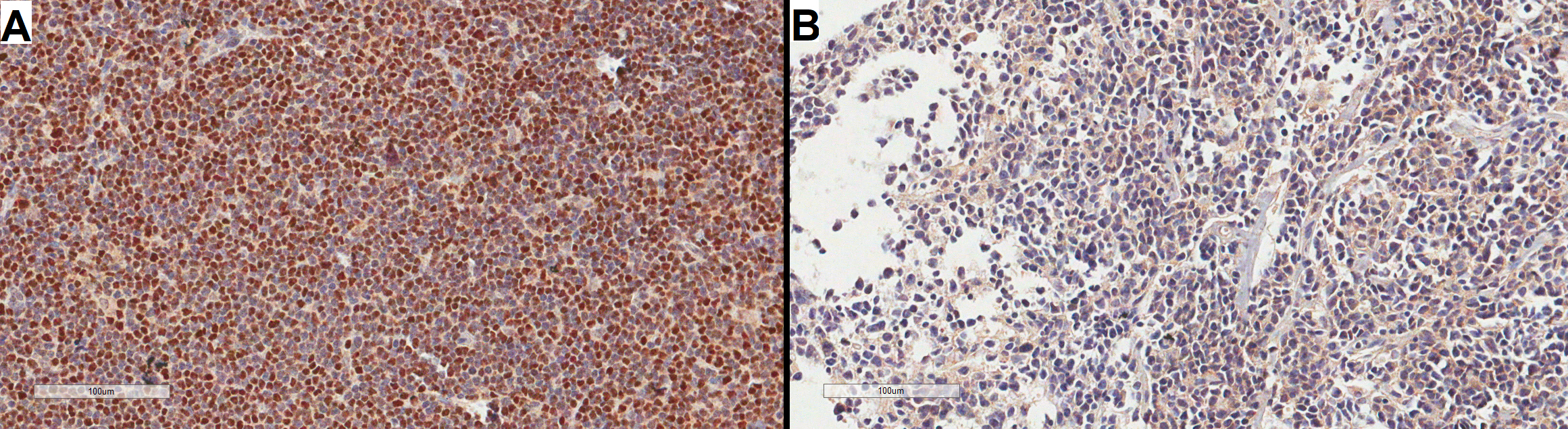

Supplement: Supplementary file 13 — Additional file 13: Supplementary Fig. 4. Representative photomicrographs of SOX11 assessment in mantle cell lymphoma. (A)SOX11high case. (B)SOX11low case. [file 12885_2021_7891_MOESM13_ESM.tif]

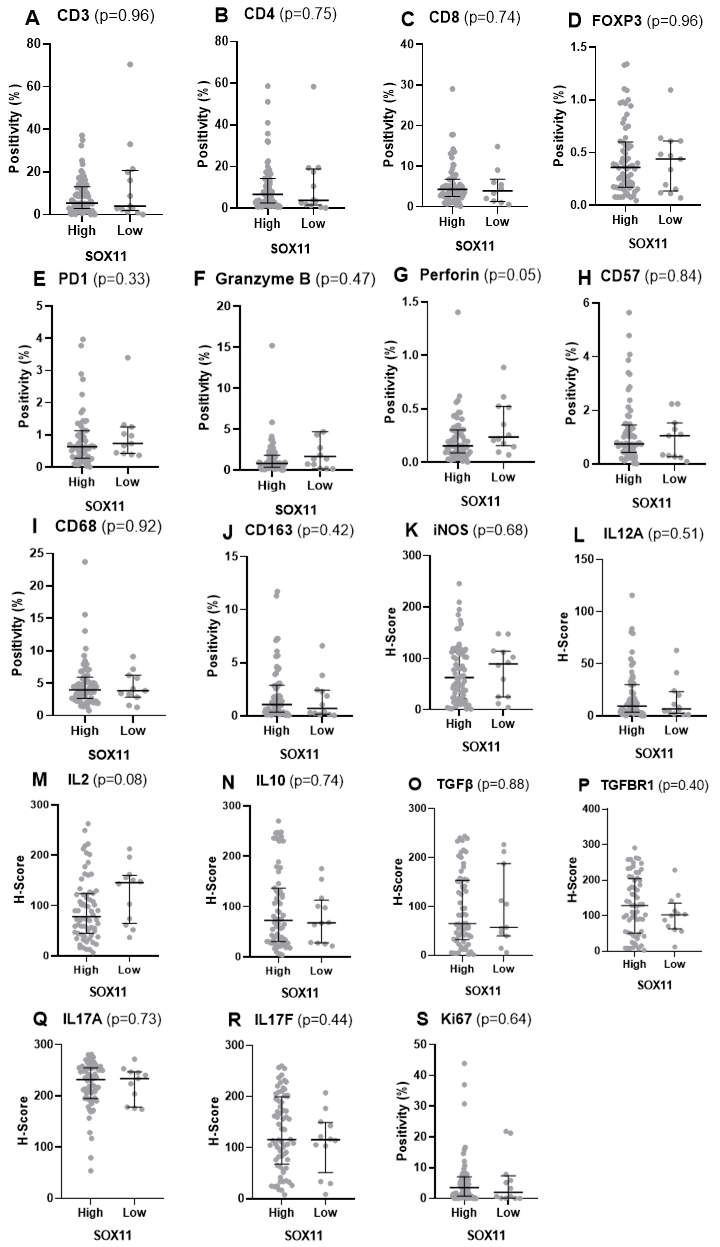

Supplement: Supplementary file 14 — Additional file 14: Supplementary Fig. 5. Tumor microenvironment markers (A-R) and proliferation index (S) in SOX11high and SOX11low mantle cell lymphoma cases. Horizontal lines represent the median levels, and the whiskers show interquartile ranges. [file 12885_2021_7891_MOESM14_ESM.tif]

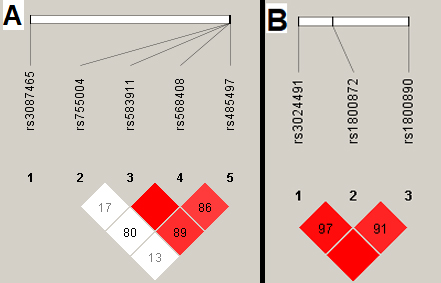

Supplement: Supplementary file 15 — Additional file 15: Supplementary Fig. 6. Linkage disequilibrium (LD) plots in mantle cell lymphoma patients for (A) IL12A and (B) IL10 genes. In each square, the LD is measured between groups of single nucleotide variants. Higher values of LD (expressed as D′) are shown in red squares. [file 12885_2021_7891_MOESM15_ESM.tif]
